# Differential expression of histone deacetylases HDAC1, 2 and 3 in human breast cancer - overexpression of HDAC2 and HDAC3 is associated with clinicopathological indicators of disease progression

**DOI:** 10.1186/1471-2407-13-215

**Published:** 2013-04-30

**Authors:** Berit Maria Müller, Lisa Jana, Atsuko Kasajima, Annika Lehmann, Judith Prinzler, Jan Budczies, Klaus-Jürgen Winzer, Manfred Dietel, Wilko Weichert, Carsten Denkert

**Affiliations:** 1 Institute of Pathology, Charité University Hospital, Campus Mitte, Charitéplatz 1, 10117, Berlin, Germany; 2 Institute of Pathology, Heidelberg, Germany; 3 Breast Cancer Center, Charité University Hospital, Campus Mitte, Charitéplatz 1 10117, Berlin, Germany; 4 Department of Pathology, Tohoku University Hospital, 1-1 Seiryo-machi, Aobaku, Sendai J980-8574, Miyagi, Japan

**Keywords:** HDAC, Breast cancer, Immunohistochemistry

## Abstract

**Background:**

In breast cancer, the role of epigenetic alterations including modifications of the acetylation status of histones in carcinogenesis has been an important research focus during the last years. An increased deacetylation of histones leads to increased cell proliferation, cell migration, angiogenesis and invasion. Class 1 histone deacetylases (HDAC) seem to be most important during carcinogenesis.

**Methods:**

The immunhistochemical expression of HDAC1, 2 and 3 was analyzed on tissue microarrays (TMAs) from 238 patients with primary breast cancer. We analyzed the nuclear staining intensity (negative, weak, moderate, strong) as well as the percentage of positive tumor cells and calculated the immunoreactivity score (0–12). Expression was correlated with clinicopathological parameters and patient survival.

**Results:**

In this cohort, we found a differential positive expression of HDAC1, HDAC2 and HDAC3. HDAC2 and HDAC3 expression was significantly higher in less differentiated tumors: HDAC2 (n=207), p<0.001 and HDAC3 (n=220), p<0.001 and correlated with negative hormone receptor status: HDAC2 (n=206), p=0.02 and HDAC3 (n=219), p=0.04. Additionally, a high HDAC2 expression was significantly associated with an overexpression of HER2 (n=203, p=0.005) and the presence of nodal metastasis (n=200, p=0.04).

HDAC1 was highly expressed in hormone receptor positive tumors (n=203; p<0.001).

**Conclusion:**

As a conclusion, our results show that the class-1 HDAC isoenzymes 1, 2 and 3 are differentially expressed in breast cancer. HDAC2 and HDAC3 are strongly expressed in subgroups of tumor with features of a more aggressive tumor type.

## Background

Despite latest individualized therapies, breast cancer is still with 14% of all estimated deaths in the United States the second leading cause of cancer related death in woman in 2012. To date, breast cancer is the most frequently diagnosed cancer in females with over 226.000 new cases [1].

During the last years, several studies about the role of epigenetic alterations including modifications of the acetylation status of histones in the development of human cancer have been published [2,3]. An increased deacetylation of histones leads to an increased cell proliferation, cell migration, angiogenesis and invasion by reducing the transcription of tumorsuppressor genes [4]. Until now, eighteen different isoenzymes of histone deacetylases (HDACs) are known which are divided into four subclasses. With respect to carcinogenesis, class 1 HDACs (HDAC1,2,3 and 8) seem to be the most important ones. HDAC1, 2 and 3 are expressed in the nucleus of normal cells and shows, in contrary to the other classes, an ubiquitous expression [5,6]. In the last years, the expression of HDACs and its prognostic value has been analyzed in different kinds of human cancers [7-9]. The prognostic role of class 1 HDACs seems to be different in various kinds of tumor entities [6]. Among the HDAC inhibitors, which can be categorized based on their structure, suberoylanilide hydroxamic acid (SAHA) was first approved for therapy for cutaneous T-cell lymphoma in 2006 [10].

The majority (70-80%) of breast cancer shows an over-expression of estrogen receptor alpha (ESR1). The endocrine therapy with first anti-estrogens or later aromatase inhibitors was one of the first targeted therapies in breast cancer, but not all of the patients with hormone receptor (estrogen and/or progesterone) positive tumors have a significant benefit due to the development of “endocrine resistance disease” [11]. In this context, a reduced activity of CYP2D6 was discussed, too [12]. The transcriptional regulation of ESR1 is influenced by multiple promoters, and acetylation was found to be one of the key mediators for transcription [13]. Recently, some authors described the effect of the addition of HDAC-inhibitors to restore the efficiency of endocrine therapy [3,14,15], for example through re-expression of ESR1 mRNA by trichostatin A or Valproate in ESR1 negative breast cancer cells [16,17]. Regarding the human epidermal growth receptor 2 (HER2), in vitro studies showed an increased degradation of HER2 after application of SAHA [18].

In this study, we analyzed the expression of the isoforms HDAC1-3 using immunohistochemical analysis on tissue microarrays (TMAs) and correlated them with relevant clinicopathological parameters, especially with hormone receptor status. Furthermore, we examined a potential prognostic impact of the expression of these proteins.

## Methods

### Study population and histopathological examination

For construction of tissue microarrays, we used formalin-fixed paraffin embedded (FFPE) tissue samples from 238 patients with primary invasive breast cancer. The overall survival was defined as the time between first diagnosis and date of death. Most of the clinicopathological data including histolocigal type, tumor size and nodal status were extracted from the pathology reports. Some parameters (grade, hormone receptor status, HER2-status) were evaluated on whole slides respectively on TMAs. The detailed patient characteristics are shown in Table 1.

**Table 1:** Patient characteristics

**Characteristic**	**No. of patients**	**%**
**All cases**	238	100
**Histological type**		
Ductal carcinoma	173	72.7
Lobular carcinoma	39	16.4
Other carcinoma	26	10.9
pT status*		
pT1	127	53.8
pT2	89	37.7
pT3	7	3
pT4	13	5.5
Nodal status*		
negative	143	63
positive	84	37
**Histological grade**		
G1	66	27.7
G2	109	45.8
G3	63	26.5
hormone receptor status*		
both negative	45	19.3
ESR1 and/or Progesterone positive	188	80.7
HER-2 status*		
negative (score 0, 1+ and 2+ SISH negative)	202	88.6
positive (score 2+ SISH positive and score 3+)	26	11.4
**age**		
<= 60 years	121	50.8
> 60 years	117	49.2

* = Not all of the data were available for all patients.

The immunhistochemical evaluation was done by a pathologist (A.K., assisted by L.J.). According to previous analyses [6] we analyzed the nuclear intensity of HDAC expression (negative, weak, moderate, strong) as well as the percentage of positive tumor cells and calculated the immunoreactivity score (IRS, 0–12) by multiplication of these two parameters. A total of 208 cases for HDAC1, 212 for HDAC2 and 224 samples for HDAC3 with expression data could be included in the final analysis. This biomarker study has been approved by the Charité University Ethics Committee (reference number EA1/139/05).

### Immunohistochemical staining

Immunohistochemical stainings were done according to standard procedures as previously described [6,9]. The following antibodies and dilutions were used: polyclonal rabbit anti-HDAC1 antibody (Abcam, Cambridge, UK; dilution: 1:11), monoclonal mouse anti-HDAC2 (Abcam, Cambridge, UK; dilution: 1:5.000), monoclonal mouse anti-HDAC3 (BD Biosience, Franklin Lakes, USA; dilution: 1:500). The specifity of the antibodies was described in previous studies [19]. After deparaffinization, the slides were boiled for 5 minutes in a pressure cooker in 0.01 M sodium citrate buffer (pH 6.0). Before incubation with the primary antibody at 4°C overnight, the slides were washed with TBS and blocked with blocking reagent for 5 to 10 minutes (Dako, Glostrup, Denmark). Subsequently, the slides were washed in TBS/Tween and the incubation with the second antibody using a streptavidin-biotin system (BioGene, San Roman, CA, USA) followed for 20 minutes at room temperature. A fast red system was used for colour developing (Sigma-Aldrich Chemie, Steinheim, Germany). At the end, the stained slides were covered with Aquatex (Merck, Darmstadt, Germany).

### Statistical evaluation

The HDAC expression was divided into three IRS groups: low (IRS 0–4), intermediate (IRS 6–8) and high (IRS 9 – 12). For statistical analysis, SPSS Statistics Version 18 (IBM, Armonk, USA) was used. P-values smaller than 0.05 were regarded as significant. χ^2^-test for trends was used for linear correlations. Survival analyses were created using Kaplan-Meier-method and log-rank test.

## Results

### Expression of HDACs in breast cancer

**Figure 1:**
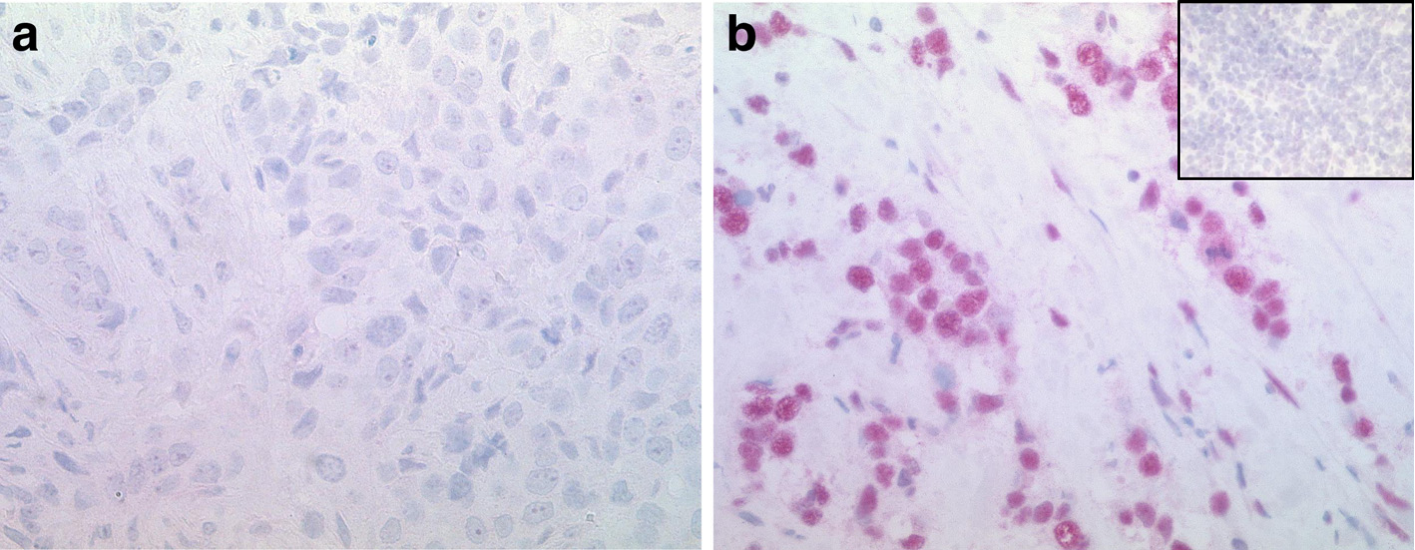
**Immunhistochemical expression of HDAC2.** Low (1**a**) expression of HDAC2 in invasive breast cancer, in this sample, only a few tumor cells (≤ 20%) show a weak HDAC2 expression (IRS 2; magnification power 400). Figure 1a shows the predominant negative expression. High (1**b**) expression of HDAC2 in invasive breast cancer, most of the tumor cells (≤ 70%) show a strong expression of HDAC2 (IRS 9; magnification power 400). The inset shows a negative expression of HDAC2 in a palatine tonsil that serves as an internal negative control (magnification power 400).

We could find a differential expression of HDAC1, HDAC2 and HDAC3 in this cohort. Most of the tumors showed an intermediate or high expression of the analysed isoenzymes. Figure 1 exemplarily depicts a breast carcinoma with a low (Figure 1a) and a high (Figure 1b) expression of HDAC2.

In breast cancer, high (IRS 9–12) nuclear expression of HDAC1, HDAC2 and HDAC3 was observed in 32.7%, 24.1% and 31.7% of cases, respectively. Low expression (IRS 0–4) of the three isoforms was found in 34.1%, 43.4% and 35.7%, whereas an intermediate expression of HDAC1, HDAC2 and HDAC3 could be seen in 33.2%, 32.5% and 32.6% of cases (Table 2).

**Table 2:** Expression of HDAC1, HDAC2 and HDAC3

**HDAC isoenzym**	**Percentage of patients with low expression**	**Percentage of patients with intermediate expression**	**Percentage of patients with high expression**
**HDAC 1**	34.1	33.2	32.7
**HDAC 2**	43.4	32.5	24.1
**HDAC 3**	35.7	32.6	31.7

### Correlation of HDAC isoforms with clinicopathological parameters

We observed significant correlations between the HDAC isoenzymes and several clinicopathological parameters.

HDAC1 was expressed higher in hormone receptor positive tumors (38.3%) vs. hormone receptor negative tumors (9.7%). Most of the hormone receptor negative cancers (53.7%) showed a low HDAC1 expression (p<0.001).

HDAC2 expression was correlated significantly with histological grade: 43.6% of the grade 3 tumors exhibited a high expression vs. 22.8% and 10% for grade 2 and grade 1 tumors, respectively (p<0.001). In contrast, 56.7% of the grade 1 tumors showed a low expression. Additionally, a high HDAC2 expression was significantly associated with a negative hormone receptor status (p=0.02) and an overexpression of HER2 (p=0.005) as well as the presence of nodal metastasis (p=0.04).

A high HDAC3 expression was observed in less differentiated (grade 3) tumors (p<0.001) and tumors with negative hormone receptor status (p=0.04). The remaining clinicopathological parameters revealed no significant correlations. The correlations of all three isoenzymes are shown in Tables 3, 4 and 5.

**Table 3:** Association of HDAC1 expression with various clinicopathological factors

**Characteristic**	**All cases**	**HDAC 1 low (IRS 0–4)**	**HDAC 1 intermediate (IRS 6–8)**	**HDAC 1 high (IRS 9–12)**	**P-value*χ***^**2**^**-test for trends**
**All cases**	208 (100%)	71 (34.1%)	69 (33.2%)	68 (32.7%)	--
**Histological type**					0.17
Ductal carcinoma/Other	176 (100%)	62 (35.2%)	60 (34.1%)	54 (30.7%)	
Lobular carcinoma	28 (100%)	7 (25%)	9 (32.1%)	12 (42.9%)	
**Histological grade**					0.89
G1	61 (100%)	24 (39.3%)	20 (32.8%)	17 (27.9%)	
G2	90 (100%)	25 (27.8%)	28 (31.1%)	37 (41.1%)	
G3	53 (100%)	20 (37.8%)	21 (39.6%)	12 (22.6%)	
**Nodal status**					0.23
negative	129 (100%)	48 (37.2%)	42 (32.6%)	39 (30.2%)	
positive	67 (100%)	18 (26.9%)	26 (38.8%)	23 (34.3%)	
**pT-Stage**					0.88
pT1	116 (100%)	41 (35.3%)	37 (31.9%)	38 (32.8%)	
pT2/pT3	78 (100%)	23 (29.5%)	30 (38.5%)	25 (32%)	
pT4	9 (100%)	4 (44.4%)	2 (22.2%)	3 (33.4%)	
**Hormone receptor status**					<0.001*
Hormone receptor positive	162 (100%)	46 (28.4%)	54 (33.3%)	62 (38.3%)	
Hormone receptor negative	41 (100%)	22 (53.7%)	15 (36.6%)	4 (9.7%)	
**HER2 status**					0.71
negative	175 (100%)	60 (34.3%)	58 (33.1%)	57 (32.6%)	
positive	25 (100%)	9 (36%)	9 (36%)	7 (28%)	

**Table 4:** Association of HDAC2 expression with various clinicopathological factors

**Characteristic**	**All cases**	**HDAC 2 low (IRS 0–4)**	**HDAC 2 intermediate (IRS 6–8)**	**HDAC 2 high (IRS 9–12)**	**P-value*χ***^**2**^**-test for trends**
**All cases**	212 (100%)	92 (43.4%)	69 (32.5%)	51 (24.1%)	--
**Histological type**					0.59
Ductal carcinoma/Other	180 (100%)	80 (44.4%)	56 (31.1%)	44 (24.5%)	
Lobular carcinoma	27 (100%)	10 (37%)	10 (37%)	7 (26%)	
**Histological grade**					<0.001*
G1	60 (100%)	34 (56.7%)	20 (33.3%)	6 (10%)	
G2	92 (100%)	39 (42.4%)	32 (34.8%)	21 (22.8%)	
G3	55 (100%)	17 (30.9%)	14 (25.5%)	24 (43.6%)	
**Nodal status**					0.04*
negative	130 (100%)	66 (50.8%)	36 (27.7%)	28 (21.5%)	
positive	70 (100%)	23 (32.9%)	28 (40%)	19 (27.1%)	
**pT-Stage**					0.43
pT1	117 (100%)	52 (44.4%)	41 (35%)	24 (20.6%)	
pT2/pT3	79 (100%)	32 (40.5%)	22 (27.9%)	25 (31.6%)	
pT4	10 (100%)	5 (50%)	3 (30%)	2 (20%)	
**Hormone receptor status**					0.02*
Hormone receptor positive	163 (100%)	73 (44.8%)	59 (36.2%)	31 (19%)	
Hormone receptor negative	43 (100%)	17 (39.5%)	7 (16.3%)	19 (44.2%)	
**HER2 status**					0.005*
negative	178 (100%)	83 (46.6%)	55 (30.9%)	40 (22.5%)	
positive	25 (100%)	5 (20%)	9 (36%)	11 (44%)	

**Table 5:** Association of HDAC3 expression with various clinicopathological factors

**Characteristic**	**All cases**	**HDAC 3 low (IRS 0–4)**	**HDAC 3 intermediate (IRS 6–8)**	**HDAC 3 high (IRS 9–12)**	**P-value *χ***^**2**^**-test for trends**
**All cases**	224 (100%)	80 (35.7%)	73 (32.6%)	71 (31.6%)	--
**Histological type**					0.34
Ductal carcinoma/Other	185 (100%)	69 (37.2%)	58 (31.4%)	58 (31.4%)	
Lobular carcinoma	35 (100%)	10 (28.6%)	12 (34.3%)	13 (37.1%)	
**Histological grade**					<0.001*
G1	63 (100%)	32 (50.8%)	19 (30.2%)	12 (19%)	
G2	101 (100%)	33 (32.7%)	35 (34.6%)	33 (32.7%)	
G3	56 (100%)	14 (25%)	16 (28.6%)	26 (46.4%)	
**Nodal status**					0.61
negative	133 (100%)	51 (38.3%)	39 (29.3%)	43 (32.4%)	
positive	78 (100%)	26 (33.3%)	26 (33.3%)	26 (33.3%)	
**pT-Stage**					0.55
pT1	119 (100%)	43 (36.1%)	37 (31.1%)	39 (32.8%)	
pT2/pT3	87 (100%)	28 (32.2%)	29 (33.3%)	30 (34.5%)	
pT4	13 (100%)	7 (53.8%)	4 (30.8%)	2 (15.4%)	
**Hormone receptor status**					0.04*
Hormone receptor positive	176 (100%)	65 (36.9%)	63 (35.8%)	48 (27.3%)	
Hormone receptor negative	43 (100%)	14 (32.6%)	7 (16.3%)	22 (51.2%)	
**HER2 status**					0.12
negative	191 (100%)	71 (37.2%)	63 (35.8%)	48 (27.3%)	
positive	25 (100%)	8 (32%)	4 (16%)	13 (52%)	

HDAC2 and HDAC3 show a strong positive correlation (p<0.001).

### Correlation of HDAC isoforms with survival

**Figure 2:**
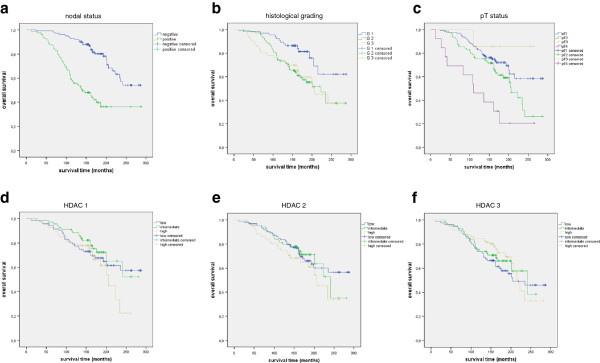
**Overall survival.** Figure 2 shows the overall survival for the whole cohort. The nodal status (Figure 2**a**; p<0.001), the histological grading (Figure 2**b**; p=0.009) as well as the pT status (Figure 2**c**; p<0.001) reached statistical significance. The expression of HDAC1 (Figure 2**d**; p=0.557), HDAC 2 (Figure 2**e**; p=0.316) and HDAC 3 (Figure 2**f**; p=0.536) did not reach significant prognostic relevance regarding the overall survival.

The known prognostic factors including nodal status (p<0.001), histopathological grading (p=0.009) and pT status (p<0.001) achieved statistical significance in this cohort. In contrast, none of the HDAC isoforms reached significant prognostic relevance in our study using Kaplan-Meyer survival analysis (Figure 2). Additionally, a co-expression of HDAC2 and HDAC3 did also not reach significant prognostic relevance (data not shown).

## Discussion

Our study demonstrates a differential expression of HDAC1, HDAC2 and HDAC3 using immunohistochemistry in breast cancer. Expression of all three isoforms revealed significant correlations with clinicopathological parameters. Expression of HDAC2 and HDAC3 was significantly higher in less differentiated tumors as well as in tumors with negative hormone receptor status. Additionally, tumors with HER2 overexpression and positive lymph node metastasis showed a significant higher expression of HDAC2. In contrast, a high expression of the HDAC1 was found in hormone receptor positive tumors.

To our knowledge, this is the first time that the class-1 isoforms HDAC1, -2 and −3 were analyzed together in the same breast cancer cohort.

Krusche et al. [20] did an immunhistochemical analysis of the expression of HDAC1 and HDAC3 in 200 breast cancer samples. Similar to our findings, they found a significant correlation between positive HDAC1 expression and positive hormone receptor expression. In contrast to our results, they additionally described a correlation of HDAC3 with a positive hormone receptor expression. They found no significant results concerning the correlation of HDAC and grading.

Similarly with our findings, Zhang et al. showed similar results concerning HDAC1, with an increased HDAC1 mRNA expression in hormone receptor positive tumors [21].

Most interestingly, we could find a significantly higher expression of HDAC2 and −3 in more aggressive tumor types. Expression of HDAC2 and −3 was higher in poorly differentiated and hormone receptor negative tumors, for HDAC2 we also found a significant correlation with HER2 overexpression. This correlation of HDACs and clincopathological parameters, which mark a more aggressive tumor type, was shown in other histological cancer types before [6].

In accordance with our results other studies might also suggest a suppression of estrogen receptor by overexpression of HDAC. Several in vitro studies analyzed the reexpression of the estrogen receptor after therapy with Trichostatin A [16]. Zhou et al. [22] achieved a restoring of estrogen receptor mRNA and protein expression. These findings suggest that estrogen receptor could be suppressed by enhanced HDAC activity and restored by HDAC inhibitors.

Additionally, multiple groups have analyzed the influence of HDAC inhibitors in estrogen receptor positive breast cancer. Here, treatment with HDAC inhibitors led to a down-regulation of estrogen receptor alpha [23,24]. In contrast, the estrogen receptor beta was shown to increase the antiproliferative potential of HDAC inhibitors as well as apoptosis as analyzed by Duong et al. [25].

In clinical studies the combination of HDAC inhibitors and hormone therapy showed first effects. Munster et al. could show an response rate of 19% for the combination of Vorinostat and Tamoxifen [26] In contrast, the monotherapy with Tamoxifen in metastatic breast cancer achieved only a response rate below 10%.

Both, in vitro and in vivo studies show that HDAC2 could be a potential biomarker. Marchion et al. showed the selective inhibition of HDAC2 in breast cancer cells to be responsible for hyperacetylation of histones and proteins [23]. In clinical studies tumors with HDAC2-expression showed a more acetylated histone status after therapy with Doxorubicin and Vorinostat [26]. HDAC2 might therefore mark tumors with response to HDAC inhibitors.

In normal mammary gland, we saw a homogenous expression of the HDAC class I isoenzymes. Similar results are described by other groups [27].

Despite our long observation time (median: 158 months) we could not observe any prognostic influence of the expression of any of the HDAC isoenzymes in this retrospective analyses. This could be due to the influence of variable therapy regimens in this time as well as the missing parameters of disease-specific deaths. Other studies have described a prognostic role for HDAC1 in breast cancer [20]. Due to the staining on a TMA, a possible heterogeneously expression of the analysed isoenzymes could be underrepresented.

Altogether, the interaction between the hormone receptor status and the HDAC expression as well as HDAC inhibitors are complex and need to be evaluated in further studies [13].

## Conclusions

As a conclusion, our results show that the class-1 HDAC isoenzymes 1, 2 and 3 are differentially expressed in breast cancer. HDAC2 and HDAC3 are strongly expressed in more aggressive tumor subtypes.

Based on our results, we suggest that HDAC inhibitors could be evaluated to restore the estrogen receptor in breast cancer cells and the combination of HDAC inhibitors and hormone therapy could be successful. Based on our results and those of other groups [10,23,28] HDAC2 might be a potential biomarker and a selective therapeutic target.

## Competing interests

The authors declare that they have no competing interests.

## Authors’ contribution

BMM arranged the tissue samples, participated in the design of the study, participated in the immunohistochemistry evaluation and draft the manuscript. LJ arranged the tissue samples, did the immunohistochemistry stainings, participated in the evaluation and in the statistical analysis. AK participated in the immunohistochemistry evaluation. AL and WW participated in the design of the study. JB did the statistical analysis. JP participated in the immunhistochemistry stainings. KJ Winzer participated in the arrangement of the cohort. MD and CD participated in its design and coordination. All authors helped to draft the manuscript, read and approved the final manuscript.

## Pre-publication history

The pre-publication history for this paper can be accessed here:

http://www.biomedcentral.com/1471-2407/13/215/prepub
